# Gaps in Education: A Cross-Sectional Survey Study of Knowledge of Advanced Lifesaving Interventions among Canadian Lifeguards

**DOI:** 10.3390/jcm13164618

**Published:** 2024-08-07

**Authors:** Riley Huntley, Connor J. O’Keefe, Filip Jaskiewicz, Klaudiusz Nadolny, Lydia Wytenbroek

**Affiliations:** 1 School of Nursing, University of British Columbia, Vancouver, BC V6T 2B5, Canada; lydia.wytenbroek@ubc.ca; 2 Department of Chemistry, McGill University, Montréal, QC H3A 0B8, Canada; connor.okeefe@mail.mcgill.ca; 3 Emergency Medicine and Disaster Medicine Department, Medical University of Lodz, 90-419 Lodz, Poland; filip.jaskiewicz@umed.lodz.pl; 4 Department of Emergency Medical Service, Faculty of Medicine, Silesian Academy in Katowice, 40-555 Katowice, Poland; prrm.knadolny@interia.pl

**Keywords:** lifeguard, oxygen administration, ventilation, drowning, first aid, education, airway management

## Abstract

**Objective:** The aim of this study was to assess lifeguards’ knowledge retention of airway management, oxygen administration, and ventilation interventions following certification and employer-provided training. **Methods:** This cross-sectional study was conducted using an online survey administered between February and May 2024. A total of 1322 responses from Canadian lifeguards certified in airway management and oxygen administration were deemed eligible for analysis. The survey included 15 knowledge assessment questions, with data analyzed based on lifeguard experience and the date of last certification or in-service training. **Results:** The mean knowledge assessment score was 10.4 ± 2.2 (69.3 ± 14.6%), with the highest scores in the airway management category and the lowest in the oxygen administration category. Lifeguard experience significantly increased knowledge retention, whereas recertification showed no significant impact, and employer-provided training significantly decreased knowledge retention. **Conclusions:** The findings underscore the importance of lifeguarding experience in knowledge retention among lifeguards. Optional airway management and oxygen administration recertification, coupled with inconsistent in-service training, have created significant gaps in lifeguard education. This study identifies the need for regular, competency-based training delivered by qualified facilitators. Addressing these gaps is crucial for enhancing the effectiveness of lifeguards in emergency response and ensuring high-quality care for drowning victims.

## 1. Introduction

Drowning is a significant global public health issue [1] and ranks as one of the leading causes of unintentional injury deaths worldwide, with an estimated 236,000 deaths annually [2]. Despite its prevalence, the true burden of drowning is often underrepresented due to inconsistent data collection and reporting, especially for nonfatal drownings, which can result in long-term disabilities and substantial economic costs [3,4,5,6]. In Canada, the incidence of fatal drownings in supervised environments is notably low, with approximately 1% of drownings occurring under lifeguard or instructor supervision [7]. Lifeguards are the first line of response in supervised aquatic environments, playing a pivotal role in drowning prevention, water rescue, and resuscitation efforts [4,8].

Lifeguard training varies significantly by country and region, affecting preparedness and effectiveness. Different regions have distinct standards and protocols, influencing the quality and comprehensiveness of training programs. This variability can impact the outcome of drowning events and resuscitation [9]. The literature indicates that well-trained lifeguards are better equipped to prevent incidents and perform timely rescues and effective resuscitation [10,11].

Lifeguards in Canada are predominately employed professionals who work in a wide variety of aquatic settings ranging from swimming pools and large waterparks to waterfronts and surf beaches. Professional lifeguard training in Canada is standardized by the Royal Life Saving Society—Canada (known as the “Lifesaving Society”). Canadian lifeguards complete a series of courses including the Bronze Medallion & Bronze Cross certification courses (40 h combined), a Standard First Aid certification course (13–16 h), and the National Lifeguard course (40 h) [12]. This pathway provides the skills, knowledge, judgement, and fitness necessary to work as a professional lifeguard in pool or waterfront environments. Additional specialized training is available for waterpark and surf environments. Lifeguards are recertified every two years by completing at least 4 h of evaluated training, commonly accompanied in BC and the Yukon with 2–4 h of optional review and updates known as “precertification”. Recertification in Standard First Aid and CPR/AED is also recommended.

Uniquely, all lifeguards in British Columbia (BC) and the Yukon participate in an Airway Management and Oxygen Administration (AMOA) course (4 h) delivered concurrently with the National Lifeguard course [12]. The requirement to have practical AMOA training can be attributed to provincial guidelines [13]. While other jurisdictions in Canada may offer lifeguards training in oxygen administration and/or airway management, training is optional and may not coincide with the National Lifeguard course. To receive AMOA certification, lifeguards must demonstrate skill proficiency through practical assessment of oropharyngeal airway (OPA) insertion, oral manual suctioning, bag valve mask (BVM) ventilation, and oxygen administration [14]. Although AMOA certification has a currency of two years, recertification is not required to maintain lifeguard certification and is rarely offered.

Lifeguard employers in Canada, most commonly municipal governments (e.g., cities), private organizations (e.g., YMCAs), and educational institutions (e.g., universities), are responsible for hiring certified lifeguards, orientating them to their workplaces, and facilitating in-service training. In-service training is a crucial component of lifeguard professional development by maintaining proficiency and confidence in emergency response [15]. These sessions reinforce theoretical knowledge and provide practical, hands-on experience essential for skill retention. It fosters a culture of continuous improvement and preparedness, allowing lifeguards to practice under simulated emergency conditions, enhancing their confidence in real-life situations [16,17,18]. Consistent with International Life Saving Federation recommendations [19], the Lifesaving Society’s national safety standard recommends training be conducted as often as possible and, at a minimum, CPR skill training should occur every 6 months [20].

Oxygen administration and BVM ventilation by trained rescuers are generally recognized as beneficial for drowning victims based on the hypoxic nature of drowning and the physiological effects of water aspiration; however, there is no direct evidence supporting its use [21,22,23,24,25]. Despite this, the use of these devices is recommended for lifeguards as non-healthcare professionals (laypersons) with a duty to respond in aquatic environments [19,26,27], provided they receive regular competency-based training to achieve and maintain proficiency [23,28,29,30].

Despite the critical importance of immediate intervention in drowning, the current literature is sparse and often inconclusive regarding the effectiveness of prehospital treatment by lifeguards [27,31]. Consequently, lifeguard training programs often rely on general guidelines based on expert consensus and physiology-driven approaches rather than data-driven practices [32,33]. This can result in inconsistent care, potentially compromising patient outcomes. Further research is needed to establish the theoretical and practical training necessary for lifeguards to achieve competency in advanced lifesaving skills such as oxygen administration, oropharyngeal airways, BVM ventilation, and manual suction [30,34]. This gap extends to the optimal frequency of training required to maintain knowledge and proficiency over time [21,26,28,35]. This study employed an online survey to assess the knowledge retention of Canadian professional lifeguards who had completed airway management and oxygen administration training.

## 2. Materials and Methods

### 2.1. Study Design

This study was designed and implemented following the Consensus-Based Checklist for Reporting of Survey Studies (CROSS) proposed by the Enhancing the QUAlity and Transparency Of health Research (EQUATOR) Network [36]. We conducted a descriptive, cross-sectional study using an online questionnaire. The design of this study was adapted from Bieliński and Jaśkiewicz [37] and influenced by previous surveys on lifeguard experience and knowledge retention [10,38,39,40].

### 2.2. Questionnaire Development

An original survey with 3 sections and 25 questions was developed using Qualtrics software version January 2024 (Qualtrics Labs Inc., Provo, UT, USA). Two additional questions were for participants employed as lifeguards or requiring current certification, and one additional question was for those who reported completion of recertification training (see Table 1). Questions related to demographic characteristics, lifeguard experience, and perceptions were identified through a selective literature review [10,37,38,39,40]. Knowledge assessment questions were derived from the Lifesaving Society’s AMOA course curriculum and were reviewed to ensure validity and reliability.

Knowledge assessment questions included multiple-choice and true or false questions. For scoring purposes, each correct question was assigned one (1) point, while incorrect or missing answers were assigned zero (0). The maximum possible score was fifteen (15). Other sections of the questionnaire included mixed question formats, including short answer, Likert-type scale responses, multiple-choice, and rank order.

### 2.3. Pretesting

Informal respondent and expert-driven pretesting was conducted using the survey before full administration. This included pretesting with 20 respondents (16 newly certified and 4 experienced lifeguards) and 2 experts (lifeguard trainers). The pretest identified that 75% of respondents could complete the questionnaire within 10 min. Survey questions were amended to provide clarity and remove ambiguous terminology. The final questionnaire is provided in File S1.

### 2.4. Sample Characteristics and Sample Size

Lifeguards were able to participate in the study if they were currently, or had previously been, certified in Airway Management and Oxygen Administration in BC and the Yukon. Convenience sampling was used to recruit potential participants through recruitment posters at aquatic facilities and training provider locations as well as by email post-certification from the certifying agency. Lifeguards certified in other provinces or territories and lifeguards who had not completed the AMOA course were excluded from the study. Participation in the survey was voluntary and anonymous.

The population size of 11,126 was established by the number of certifications issued since the AMOA course launch in 2021. Using a 95% confidence level and a 5% error margin, the minimum sample size was 372. The survey was successfully delivered to 9985 lifeguards by email and supported by recruitment posters, resulting in 1329 total responses. Six responses were ineligible due to missing data and one response was discarded for ballot stuffing. With 1322 complete responses, an error margin of 2.5% and a response rate of 13.2% was achieved. The sample characteristics are consistent with the population, aligning with an internal Lifesaving Society research report dated 2009 [41].

### 2.5. Survey Administration

Ethical approval was granted by the Behavioural Research Ethics Board (H24-00014) at the University of British Columbia. Informed consent was obtained from participants. Participants were advised of the anticipated completion time of 10–15 min, that the research findings would be submitted for publication in a research journal, and that the research data were anonymized. The investigators and purpose of the study were disclosed to participants in the survey cover letter. A chance to win one of five lifeguard tool kits (valued at $69) was offered as an incentive for participation. A total of 1172 (88.7%) of 1329 respondents opted to take part in the incentive raffle draw. This suggests that the incentive appealed to respondents, highlighting the significance of additional motivation in boosting survey participation.

The survey was administered online through Qualtrics software between 15 February and 5 May 2024. For participants contacted by email, one follow-up email was sent to participants who had not completed the survey within two weeks. Qualtrics’ bot and duplicate-response detection features were used to assess submission validity. Flagged submissions were reviewed by the investigators and one duplicate response was discarded.

### 2.6. Procedures

Participants were requested to complete the survey independently without referencing any course materials or other sources of information (e.g., web search, instructors, coworkers, etc.). They were encouraged to share the survey with other qualified lifeguards, but not to discuss the questions with others who had not yet completed the survey.

### 2.7. Data Analysis

Data were collected using Qualtrics software and statistical analysis was performed using RStudio version 4.3.3 (R Foundation for Statistical Computing, Vienna, Austria) and Qualtrics Stats iQ statistical software version May 2024. Demographic scores were analyzed using descriptive statistics; additional numerical variables are reported as mean with standard deviation or median with interquartile range as appropriate.

One-way ANOVA testing was performed when assessing the impact of categorical variables on knowledge assessment scores. In cases where multiple categorical variables could affect the assessment score, two-way ANOVA testing was performed to verify results. Normality of the residuals was tested by examining normalized Q-Q plots and fitted residual histograms; all reported data follow normality, eliminating the need for nonparametric testing. To assess relationships between continuous ordinal variables, Pearson’s correlation coefficient is reported with a 95% confidence interval. The significance level for all tests was predetermined to be *p* < 0.05.

Six questionnaires missing one-third (5) or more of the questions (data missing completely at random) in the knowledge assessment section were excluded. A total of 14 questionnaires with fewer than one-third (5) of questions answered were assigned a zero on missing questions. For all other sections, any missing data (<2%, within the margin of error) were reported as “Prefer not to answer” or omitted from the analysis.

## 3. Results

A total of 1322 lifeguards were included in the study.

### 3.1. Lifeguard Demographics, Experience, and Training/Certification

The mean age of lifeguards was 23 ± 10 (M = 19 ± 7), with the majority under 21 years of age (59.9%, *n* = 786). The majority of lifeguards were female (57.6%), employed in a role requiring current lifeguard certification (69.8%), and more than half of lifeguards (58.6%) had 2 years or less of volunteer or professional experience (Table 2).

### 3.2. Knowledge Assessment

The mean score of the 15-question knowledge assessment was 10.4 ± 2.2 (69.3 ± 14.6%). The mean scores (±SD) per 5-question category were as follows: airway management: 3.7 ± 1.0 (74 ± 20%), oxygen administration: 3.2 ± 1.2 (64 ± 24%), and ventilation: 3.6 ± 1 (72 ± 20%). Question topics and the frequency of correct answers are presented in Table 3. File S1 includes the questionnaire with the original knowledge assessment questions. There is a weak positive relationship between total score and age (ρ = 0.2; 95% CI [0.19, 0.29]). Lifeguards who reported current employment as a lifeguard or in a role requiring certification (*n* = 923) had a mean overall score of 10.6 ± 2.2 (70.7 ± 14.7%). A total of 32 lifeguards received 15/15 (100%) on the knowledge assessment.

Knowledge retention of oxygen administration was weakest amongst the three categories. Analysis of the questions concerning oxygen administration identified that lifeguards struggle to determine the clinical indications of administrating oxygen. The frequency of correct answers was notably low for administering oxygen based on hypoxic conditions requiring immediate oxygen (52.9%) and oxygen saturation assessed by pulse oximetry (57.6%). Likewise, a significant number of lifeguards believed that there is no risk of harm by administering oxygen to victims who do not require it (53.3%).

**Figure 1:**
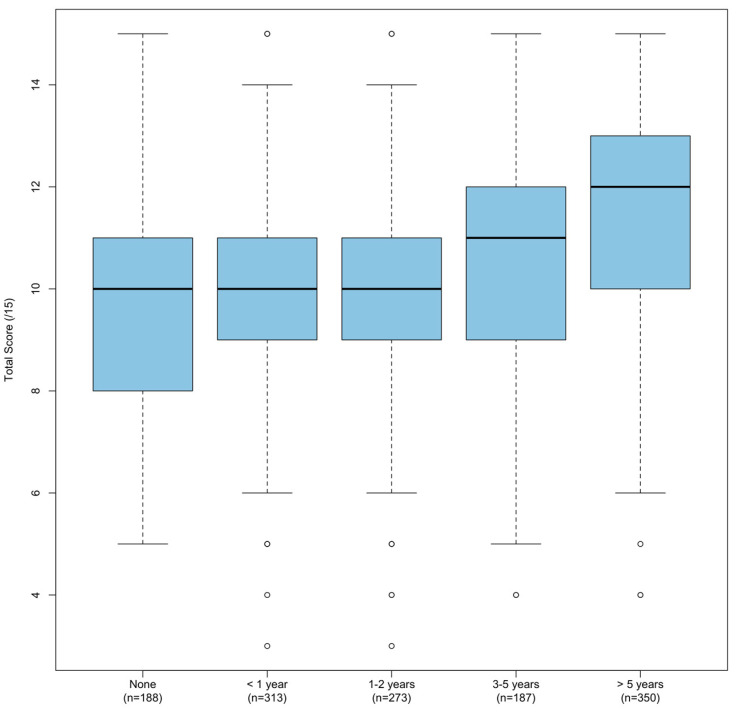
Boxplot of correct answers (total score), grouped by years of experience as a lifeguard.

The most consistent predictor of total score was found to be years of lifeguarding experience (*p* < 0.001), with total score consistently increasing with experience (Figure 1 and Table 4).

**Figure 2:**
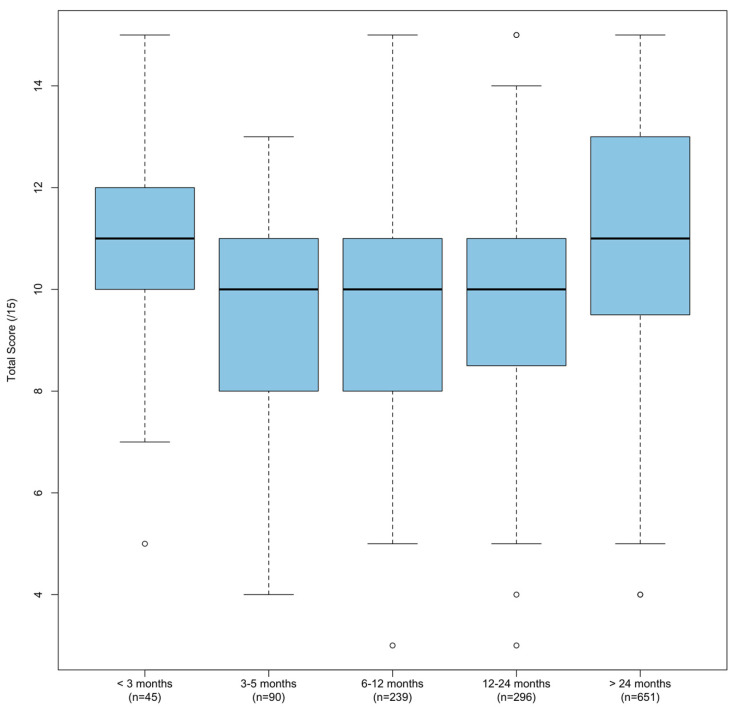
Boxplot of correct answers (total score), grouped by months passed since initial lifeguard certification.

Time since the original lifeguard training course also had a significant (*p* < 0.001) impact on the total score (Figure 2 and Table 5). However, in this case, the total score sharply decreased between less than 3 months and 3–5 months and increased as time increased (*p* < 0.001, excluding < 3 months).

Neither time since the most recent employer in-service or orientation training (*p* = 0.869) nor time since the most recent recertification course (*p* = 0.497) had a statistically significant impact on total score. To account for confounding variables due to the correlation of recertification and experience, two-way ANOVA testing was performed. Length of experience lifeguarding and time since most recent recertification were both analyzed. The results showed a significant difference in the total score when recertification time was held constant and experience varied (*p* < 0.001). However, there was no significant difference (*p* = 0.483) when recertification was varied with experience held constant. Similar results were observed when adjusting for experience in relation to the time since most recent employer-provided in-service or orientation training.

**Figure 3:**
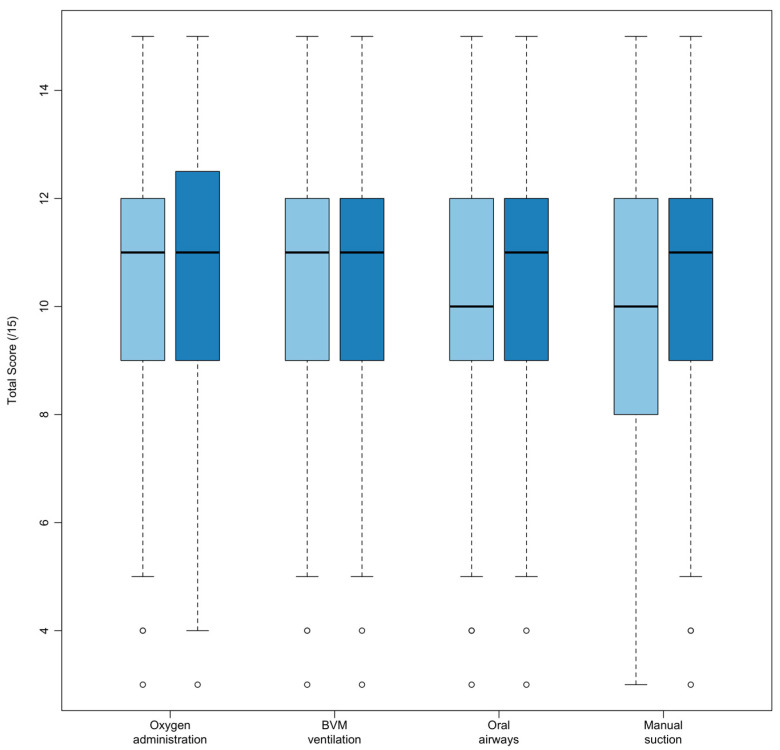
Boxplot of correct answers (total score), filtered for lifeguards that attended employer-provided training within the past year. Scores are grouped by whether the most recent in-service or orientation included an AMOA skill (light blue) or if the training did not include the skill (dark blue).

Lifeguards who most recently attended employer-provided training that included AMOA content performed significantly worse compared to training that did not include AMOA content (Figure 3 and Table 6).

After adjusting for employer-provided training conducted more than one year ago as a confounding variable, training of oral airways and manual suction had statistically significant total score decreases, whereas training of oxygen administration and bag valve mask ventilation had statistically insignificant total mean score decreases (Table 6).

### 3.3. Excluded Questions

In this study, five survey questions measuring experience and perception were excluded from data analysis. Two of these questions related to personal experience and three of the questions related to learning preferences. These questions were designed to test a separate hypothesis within the context of curriculum development. Secondary findings will therefore be published separately, allowing for a more detailed and rigorous analysis.

## 4. Discussion

The principal findings of this study show that length of lifeguard experience significantly influences knowledge retention in airway management, oxygen administration, and ventilation. The results also indicate that proximity to lifeguard recertification and employer-provided training do not significantly impact knowledge retention when adjusted for lifeguard experience, suggesting that current training practices may be less effective than anticipated. This underscores the need for more focused training programs to ensure that lifeguards maintain critical lifesaving skills.

The results presented in Table 5 show a clear pattern in lifeguards’ knowledge retention over time following initial certification. Lifeguards scored highest during the first three months, indicating that training knowledge was still fresh. A significant drop in the 3–5-month period indicates rapid knowledge decline, followed by a marginal increase in the 6–12-month period, maintained during the 1–2 years period, suggesting stabilization but not a full recovery. A significant increase after two or more years suggests that long-term practical experience and repeated application of skills enhance knowledge retention. This pattern highlights the importance of ongoing practical experience and frequent refresher training within the first 2 years post-certification to maintain competency. The authors considered whether changes in training curricula could explain score variances; however, no significant changes occurred.

Concerns were identified regarding lifeguards’ understanding of oxygen administration. Lifeguards receive theoretical and practical training in the AMOA course on the clinical indications for administering oxygen based on select hypoxic conditions or pulse oximetry to minimize potential harm [42]. This aligns with evidence suggesting that, while there is no harm in prehospital oxygen administration for victims experiencing respiratory distress or hypoxia (e.g., nonfatal drowning), routine oxygen administration can lead to adverse outcomes for certain patient groups (e.g., acute myocardial infarction, stroke, etc.) when not indicated [21,43,44]. Despite their training, lifeguards struggled to identify clinical indications for administering oxygen and recognize the harm of unnecessary oxygen administration. These findings highlight a critical gap in knowledge, with the potential to impact quality of care [23]. Training should emphasize accurate assessment of hypoxia, proper use of pulse oximetry, and understanding the risks of routine oxygen administration. Addressing these gaps is essential to ensure that lifeguards can make informed decisions during emergencies.

Consistent with the recommendation of organizations such as the International Life Saving Federation and the International Liaison Committee on Resuscitation [21,26], the results of the study highlight the value of regular competency-based training [21,28]. In a study similar to this, Bieliński and Jaśkiewicz [37] suggest that poor knowledge retention despite recertification training is the result of “ineffective recertification and its rarity of occurrence”. The optional nature and lack of adoption result in rare AMOA recertification within BC and the Yukon, preventing lifeguards from receiving consistent competency-based training.

Despite the majority (56.8%) of employed lifeguards participating in employer training in the last 3 months, a significant number of lifeguards reported that training did not include manual suction (70.7%), oral airways (39.4%), BVM ventilation (39.8%), or oxygen administration (31.5%). Employers are encouraged to reassess the frequency with which their lifeguards receive training containing AMOA content. It is concerning that when AMOA content was included, knowledge assessment scores showed either no increase (oxygen administration and BVM ventilation) or a statistically significant decrease (oral airways and manual suction). Employers should consider the qualifications of training facilitators and the integration of recognized training materials to reduce quality concerns. The knowledge assessment was designed to assess content mastery, comprising mandatory content from the AMOA curriculum. Since any part of the content could be used in a real-life rescue, the poor results (mean score of 70.7 ± 14.7%) of currently employed lifeguards further underscore the necessity of enhanced training, as current methods leave noticeable gaps in lifesaving program content.

The principles of the Utstein Formula for Survival (FfS) can be applied in the context of lifeguard resuscitation training to highlight quality guidelines (science), efficient training (education), and effective integration (local implementation) as factors that can improve cardiac arrest survival [9,45]. The addition of oxygen, bag valve masks, airways, or any other intervention to the lifeguard toolkit will not increase the survival of drowning alone. Certifying agencies and lifeguard employers must understand that successful resuscitation extends beyond the critical moment of resuscitation itself [29]. Ultimately, the FfS concept outlines that survival is driven by medical science, education, and local implementation [9,25]. This study illustrates that agencies and employers share the responsibility of producing effective lifeguards through a collaborative approach that combines foundational education and professional development. This responsibility extends to consideration of the full spectrum of factors that contribute to resuscitation outcomes, which should be addressed prior to implementing advanced lifesaving interventions. This holistic approach ensures that lifeguards are not only equipped with the necessary tools, but also possess the knowledge and skills to use them effectively in real-world scenarios.

One of the notable strengths of this study in comparison to other surveys is its large sample size, which enhances the generalizability and robustness of the findings. The extensive participation provides a comprehensive understanding of knowledge retention among lifeguards in airway management, oxygen administration, and ventilation. Additionally, the design of the survey gives fresh consideration to the potential influence of employer in-service and orientation training on reinforcing knowledge retention. Despite the large sample size, the survey was confined to BC and the Yukon due to the inconsistent availability of AMOA training in Canada, potentially limiting the applicability of the findings to other regions. The AMOA curriculum, while consistent with other lifeguarding programs, lacks an evidence-based foundation due to a general shortage of drowning research. In addition to established research gaps in oxygen administration and bag valve mask ventilation, the effectiveness of oropharyngeal airways and manual suction in drowning or cardiac resuscitation by lifeguards is not well studied [33]. Certifying agencies should critically analyze existing AMOA training models before adoption to ensure practices align with evidence-based research and quality guidelines.

Similar to previous studies, unanswered questions remain regarding the optimal frequency and content of recertification needed to maximize knowledge retention and training quality. A research gap was identified during the literature review when a recommended minimum threshold or standard (e.g., “passing score”) for knowledge assessments could not be identified. Future research should evaluate the effectiveness of employer-provided training for advanced interventions and its impact on long-term knowledge retention and practical skills. Studies involving practical assessments or simulations under stress could provide deeper insights into the actual preparedness of lifeguards in real-life situations.

## 5. Conclusions

The findings of this study reveal significant gaps in the knowledge retention of airway management and oxygen administration among Canadian lifeguards, underscoring the critical influence of lifeguarding experience on maintaining these essential skills. The findings highlight the limited impact of recertification and employer-provided training on knowledge retention, suggesting that current training practices may not adequately reinforce these life-saving skills. Notably, lifeguards with greater professional experience demonstrated higher knowledge retention, emphasizing the importance of practical exposure over time. This study underscores the need for more effective and standardized training programs, as highlighted by the Formula for Survival, which emphasizes quality guidelines, efficient training, and effective integration to improve emergency response outcomes. Addressing these gaps is crucial for enhancing the effectiveness of lifeguards in providing high-quality emergency care.

## 6. Limitations

The study was confined to lifeguards in BC and the Yukon, the only province and territory of Canada with universal adoption of AMOA training. The inconsistent availability of AMOA training in other provinces and territories posed a risk to the internal validity of the study due to participant bias. However, the AMOA program’s comprehensive curriculum is similar to airway management and oxygen administration training available in Canada and internationally, supporting the generalizability of the study results. While consistent with previous lifeguard knowledge retention studies [10,37,39,40], the conventional scoring mechanism of the questionnaire did not permit the assessment of partial knowledge. Partial credit could impede the study’s design of assessing mastery of knowledge and obscure the identification of gaps in full comprehension.

## Figures and Tables

**Table 1:** Structure of the final resulting questionnaire.

Section/Subsection	Number of Questions
Sociodemographic characteristics	5–8
Non-employed lifeguard	5
Employed lifeguard	2
Recertified lifeguard	1
Knowledge assessment	15
Airway management	5
Oxygen administration	5
Ventilation	5
Lifeguard experience and perceptions	5
Experience and confidence	2
Training frequency, duration, and resources	3

**Table 2:** Sociodemographic characteristics of participants. Questions left blank were included in “Prefer not to answer”.

Characteristics (*N* = 1322)	*n*	%
Gender		
Female	762	57.6
Male	516	39.0
Nonbinary	20	1.5
Prefer not to answer	24	1.8
Age		
15–17 years	416	31.5
18–20 years	370	28.0
21–25 years	248	18.8
26–35 years	134	10.1
≥36 years	145	11
Prefer not to answer	9	0.7
Employment status		
Lifeguard or role requiring lifeguard certification	923	69.8
Unrelated employment	203	15.4
Unemployed	178	13.5
Prefer not to answer	18	1.4
Lifeguard experience		
No experience	188	14.2
<1 year	313	23.7
1–2 years	273	20.7
3–5 years	187	14.1
>5 years	350	26.5
Prefer not to answer	11	0.8
Time since initial certification		
<3 months ago	45	3.4
3–5 months ago	90	6.8
6–12 months ago	239	18.1
1–2 years ago	296	22.4
>2 years ago	651	49.2
Prefer not to answer	1	0.1
Time since lifeguard recertification	*n* = 649	
<3 months ago	77	11.9
3–5 months ago	71	10.9
6–12 months ago	190	29.3
1–2 years ago	218	33.6
>2 years ago	93	14.3
Time since last employer in-service/orientation	*n* = 922	
<3 months ago	524	56.8
3–5 months ago	218	23.6
6–12 months ago	137	14.9
1–2 years ago	30	3.3
>2 years ago	13	1.4

**Table 3:** Frequency of correct answers to the individual questions in the knowledge assessment.

#	Question Topic (*N* = 1322)	Correct Response	
		*n*	%
1	Airway: OPA sizing	1200	90.8%
2	Airway: Agonal breathing	1149	86.9%
3	Ventilation: Supplemental oxygen	1136	85.9%
4	Ventilation: Two-rescuer BVM	1118	84.6%
5	Airway: Gastric distension	1105	83.6%
6	Airway: OPA indications	1032	78.1%
7	Oxygen: Delivery devices	1017	76.9%
8	Oxygen: Flow rate	991	75.0%
9	Airway: Obstruction	914	69.1%
10	Ventilation: Rescuer positioning	771	58.3%
11	Oxygen: Pulse oximetry	762	57.6%
12	Oxygen: Risk of harm	705	53.3%
13	Oxygen: Hypoxia	699	52.9%
14	Ventilation: Inspiratory time	601	45.5%
15	Airway: Suction visualization	583	44.1%

**Table 4:** Comparison of correct answers (total score) in study group, grouped by professional experience.

Professional Lifeguard Experience (*N* = 1311)	*x* (*SD*)	*M* (*IQR*)	*n*
No experience	9.7 (2.2)	10 (3)	188
<1 year	9.9 (2.1)	10 (2)	313
1–2 years	10.0 (2.2)	10 (2)	273
3–5 years	10.5 (2.2)	11 (3)	187
>5 years	11.5 (2.1)	12 (3)	350
*p*	<0.001		

*x*—mean; *SD*—standard deviation; *M*—median; *IQR*—interquartile range.

**Table 5:** Comparison of correct answers (total score) based on time passed since initial certification.

Time Passed Since Initial Certification (*N* = 1311)	*n*	*x* (*SD*)	*M* (*IQR*)
<3 months	45	11.0 (2.1)	11 (2)
3–5 months	90	9.7 (2.2)	10 (3)
6–12 months	239	9.9 (2.1)	10 (3)
1–2 years	296	9.9 (2.2)	10 (2.25)
>2 years	651	11.0 (2.2)	11 (3.5)
*p*	<0.001		

*x*—mean; *SD*—standard deviation; *M*—median; *IQR*—interquartile range.

**Table 6:** Comparison of correct answers (total score) of lifeguards that attended employer-provided training within the past year grouped by skill covered during the most recent in-service or orientation.

Skill	*n*	Skill Included *x* (*SD*)	Skill Not Included *x* (*SD*)	*p*-Value
Oxygen administration	739	10.4 (2.2)	10.7 (2.4)	0.17
Bag valve mask devices	749	10.6 (2.1)	10.6 (2.3)	0.58
Oral airways	738	10.4 (2.2)	10.8 (2.2)	0.015
Manual suction	781	9.9 (2.3)	10.8 (2.2)	<0.001

*x*—mean; *SD*—standard deviation.

## Data Availability

The raw data supporting the conclusions of this article will be made available by the authors on request.

## Supplementary Materials

The following supporting information can be downloaded at: https://www.mdpi.com/article/10.3390/jcm13164618/s1, Supplementary File S1: Survey questionnaire form; Supplementary Table S1: Checklist for Reporting Of Survey Studies (CROSS) Reporting Results.
