# Supplementary material for: Gaps in Education: A Cross-Sectional Survey Study of Knowledge of Advanced Lifesaving Interventions among Canadian Lifeguards
Source: J Clin Med. 2024 Aug 7;13(16):4618. doi: 10.3390/jcm13164618 (PMC11354855; doi:10.3390/jcm13164618)
Supplement: Supplementary file 1 [file jcm-13-04618-s001.zip › jcm-3148435-supplementary.pdf]

Supplement 1: Online Survey

Lifeguard AMOA Survey

We are conducting research to help answer questions about **lifeguard knowledge retention and perceptions of airway management and oxygen administration (AMOA).**

This is a short online survey designed to give us insight into the knowledge retained by lifeguards and their perceptions following completion of the provincial AMOA course. This course is embedded in all lifeguard courses delivered in British Columbia and the Yukon. It is our aim to get a clearer understanding of how well lifeguards retain information delivered in the provincial AMOA course, the rate at which lifeguards' knowledge downtrends over time, and their perceived abilities and experience to use AMOA techniques and devices. The survey contains questions on:

- Participant demographics (employment status and experience)
- Airway management knowledge assessment
- Oxygen administration knowledge assessment
- Ventilation knowledge assessment
- Experience and perceived ability to use this knowledge

After completion of the study, you will be redirected to an independent page where you can enter a draw to win 1 of 5 – Lifesaving Society Lifeguard Tool Kits valued at $69. This includes a branded fanny pack, whistle, super scissors, a pocket mask, and a set of 6 airways. The personal information (such as your name and email address) you provide for the draw will be saved in a password-protected encrypted spreadsheet and will be stored separately from the main survey.

The research findings will be submitted for publication in a research journal. As the research data is anonymized, there is no increased risk for participants. Once this data is published, you cannot withdraw from the study.

Eligibility

To be eligible for the study, you must:

- be a Canadian lifeguard; and
- be currently or previously certified in British Columbia or the Yukon.

Risks and Benefits

- The risks of participating are not greater than the risks of everyday life.
- There are no direct benefits to the participants for being in the study. By entering the draw (optional), participants will have the opportunity to win a prize.

Confidentiality

- There are no questions in the main survey that ask for directly identifying information (e.g. name, certification number, etc.).
- If you choose to enter the optional draw, personal information is collected in a separate survey for the purposes of conducting the draw.

Voluntary Participation and Freedom to Withdraw

- It is your choice to be in this study. Your participation is optional.
- You can withdraw from the study by contacting the research team with your response ID. Once the research findings are published, you cannot withdraw.
- Everyone who participates in the study, including those who withdraw, will have the opportunity to enter the draw.

Questions or Concerns

If you have questions or desire further information with respect to this study, you should inquire with Dr. Lydia Wytenbroek at Lydia.Wytenbroek@ubc.ca or Riley Huntley at RileyDJ@student.ubc.ca. If you have any concerns

or complaints about your rights as a research participant and/or your experiences while participating in this study, contact the Research Participant Complaint Line in the UBC Office of Research Ethics at 604-822-8598 or if long distance e-mail RSIL@ors.ubc.ca or call toll free 1-877-822-8598

Participating in the Study
We anticipate it will take ~10-15 minutes to complete the survey, somewhat dependent on your experience. By clicking the button below, you acknowledge that your participation in the study is voluntary and that you are aware that you may choose to terminate your participation in the study at any time and for any reason.

Answer as best as you can without referencing any course materials or other sources of information (e.g. Google, instructors, classmates, or coworkers, etc.). You may share this survey with peers but are requested not to discuss the questions with others who have not yet completed the survey.

○ I consent, begin the study

○ I do not consent, I do not wish to participate

End of Block: Informed Consent

Start of Block: Demographics

Lifeguard Demographic Questions

Q1 How old are you?

____

Q2 What is your gender identity?

○ Woman

○ Man

○ Non-binary person

○ Prefer not to answer

Q3 Which statement best describes your current employment status?

○ Employed as a lifeguard or in a role requiring current lifeguard certification

○ Employed in any other role

○ Not employed

○ Prefer not to answer

Q4 When did you complete your original National Lifeguard **full** course?

- Less than 3 months ago
- 3 - 5 months ago
- 6 - 12 months ago
- 1 - 2 years ago
- More than 2 years ago

Display This Question:

If Q4 = More than 2 years ago

Q5 When did you complete your last National Lifeguard **recertification** course?

- Less than 3 months ago
- 3 - 5 months ago
- 6 - 12 months ago
- 1 - 2 years ago
- More than 2 years ago (expired)

Display This Question:

If Q3 = Employed as a lifeguard or in a role requiring current lifeguard certification

Q6 When was your most recent lifeguard orientation or inservice training?

- Less than 3 months ago
- 3 - 5 months ago
- 6 - 12 months ago
- 1 - 2 years ago
- More than 2 years ago

Display This Question:

If Q3 = Employed as a lifeguard or in a role requiring current lifeguard certification

Q7 During your most recent lifeguard inservice or orientation, which of the following skills were covered:

	Yes	Somewhat	No
Oxygen administration	○	○	○
Bag valve mask devices	○	○	○
Oral airways	○	○	○
Manual suction	○	○	○

Display This Question:

If Q4 = More than 2 years ago

Q8 How many years of lifeguarding experience do you have?

○ Less than 1 year

○ 1 - 2 years

○ 3 - 5 years

○ Greater than 5 years

End of Block: Demographics

Start of Block: Airway management

Airway management questions (5)

Answer as best as you can without referencing any course materials or other sources of information (e.g. Google, instructors, classmates, or coworkers, etc.).

Knowledge assessment section of the questionnaire, in order. Correct answers based on certifying program are bolded.

Q1 Using the AVPU assessment tool, identify what type of victims oral airways are inserted in:

- A - Alert
- V - Verbal response
- P - Pain response
- **U - Unresponsive**
- Not familiar with the AVPU assessment tool

Q2 The largest size oral airway in the oxygen kit should be inserted to ensure an adequate airway:

- True
- **False**

Q3 The advantage of manual suction is being able to suction the airway beyond what you can see:

- True
- **False**

Q4 Which of the following is the most common cause of airway obstruction in an unresponsive victim?

- **The tongue**
- Vomit or bodily fluid
- Food
- Foreign objects

Q5 Victims of sudden cardiac arrest may present "agonal breathing" in the first few minutes after collapse. Agonal breathing is best described as:

- **Gasping and sporadic breathing**
- Coughing and rapid breathing
- Deep and long breaths
- Absent breathing

End of Block: Airway management

Start of Block: Oxygen Administration

Oxygen Administration Questions (5)

Q0 Answer as best as you can without referencing any course materials or other sources of information (e.g. Google, instructors, classmates, or coworkers, etc.).

Knowledge assessment section of the questionnaire, in order. Correct answers based on certifying program are bolded.

Q1 Oxygen should be administered to victims at what oxygen saturation level?

- Less than (<) 95%
- **Less than (<) 94%**
- Less than (<) 92%
- Less than (<) 90%

Q2 Which of the following is a condition in which supplemental oxygen is **not** immediately administered?

- Drowning
- Carbon monoxide poisoning
- **Stroke**
- Decompression illness

Q3 The recommended oxygen flow rate for a non-breathing victim is 10 litres per minute:

- True
- **False**

Q4 Which oxygen delivery device would be used for a non-breathing victim?

○ Non-rebreathing mask

○ **Bag valve mask**

○ Standard face mask

○ Nasal cannula

Q5 Administering oxygen to victims that do not require oxygen may cause harm

○ **True**

○ False

End of Block: Oxygen Administration

Start of Block: Ventilation

Ventilation Questions (5)

Answer as best as you can without referencing any course materials or other sources of information (e.g. Google, instructors, classmates, or coworkers, etc.).

Q1 How long should you squeeze the bag for when ventilating a victim with a bag valve mask (BVM) device?

○ Less than 1 second

○ **1 second**

○ 2 seconds

○ More than 2 seconds

Q2 A bag valve mask device can be used without being connected to oxygen

○ **True**

○ False

Q3 Which of the following is most likely to cause gastric distention when ventilating with a bag valve mask?

- Poor mask seal
- **Squeezing the bag too much**
- Delivering ventilations too slowly
- Using an oral airway

Q4 Where should the rescuer responsible for sealing the mask of the BVM be positioned?

- On either side of the victim
- **At the head of the victim**
- Either of the above are acceptable

Q5 During two-rescuer CPR, which rescuer is responsible for sealing the mask of the BVM?

- The rescuer performing compressions
- **The rescuer opening the airway**
- Either of the above are acceptable

End of Block: Ventilation

Start of Block: Lifeguard Experience and Perceptions

Lifeguard Experience and Perceptions (5)

Q1 Have you used any of the following devices in a real life-threatening emergency?

	Yes	Somewhat	No
Oxygen administration	○	○	○
Bag valve mask devices	○	○	○
Oral airways	○	○	○
Manual suction	○	○	○

Q2 Do you feel confident in the being able to effectively use these devices during drowning resuscitation?

	Yes	Somewhat	No
Oxygen administration	○	○	○
Bag valve mask devices	○	○	○
Oral airways	○	○	○
Manual suction	○	○	○

Q3 How often do you think employed lifeguards should receive hands-on training in these devices?

○ Every 6 months

○ Every year

○ Every two years

○ Every three years

Q4 How long do you feel this hands-on training should be?

○ Less than 1 hour

○ 1 - 2 hours

○ 3 - 4 hours

○ More than 4 hours

Q5 Rank the following learning resources in order of preference for refreshing or maintaining knowledge: 1 being your most preferred form and 5 being your least preferred form.

_______ YouTube videos
_______ Electronic manual
_______ Paper/printed manual
_______ eLearning/online modules
_______ Candidate workbook

End of Block: Lifeguard Experience and Perceptions

Start of Block: Raffle

Raffle (1)

Q1 Would you like to enter the raffle to win a prize? Your response will still remain anonymous.

○ Yes

○ No

End of Block: Raffle

Start of Block: ResponseID

Your response has been recorded anonymously. If you wish to withdraw your response from this study, contact the research team providing the response ID: **{ResponseID}**. This number is unique to your response. Please note this for your records now if you think you may want to withdraw your response in the future.

Select the "**Enter Raffle**" button, in the bottom right corner, when ready to be redirected to the raffle page.

End of Block: ResponseID

Table S1: Checklist for Reporting Of Survey Studies (CROSS)

Section/topic	Item	Item description	Reported on page #
**Title and abstract**			
Title and abstract	1a	State the word “survey” along with a commonly used term in title or abstract to introduce the study’s design.	1
	1b	Provide an informative summary in the abstract, covering background, objectives, methods, findings/results, interpretation/discussion, and conclusions.	1
**Introduction**			
Background	2	Provide a background about the rationale of study, what has been previously done, and why this survey is needed.	1-2
Purpose/aim	3	Identify specific purposes, aims, goals, or objectives of the study.	2
**Methods**			
Study design	4	Specify the study design in the methods section with a commonly used term (e.g., cross-sectional or longitudinal).	2
Data collection methods	5a	Describe the questionnaire (e.g., number of sections, number of questions, number and names of instruments used).	2-3
	5b	Describe all questionnaire instruments that were used in the survey to measure particular concepts. Report target population, reported validity and reliability information, scoring/classification procedure, and reference links (if any).	3
	5c	Provide information on pretesting of the questionnaire, if performed (in the article or in an online supplement). Report the method of pretesting, number of times questionnaire was pre-tested, number and demographics of participants used for pretesting, and the level of similarity of demographics between pre-testing participants and sample population.	3
	5d	Questionnaire if possible, should be fully provided (in the article, or as appendices or as an online supplement).	S1
Sample characteristics	6a	Describe the study population (i.e., background, locations, eligibility criteria for participant inclusion in survey, exclusion criteria).	3
	6b	Describe the sampling techniques used (e.g., single stage or multistage sampling, simple random sampling, stratified sampling, cluster sampling, convenience sampling). Specify the locations of sample participants whenever clustered sampling was applied.	3
	6c	Provide information on sample size, along with details of sample size calculation.	3
	6d	Describe how representative the sample is of the study population (or target population if possible), particularly for population-based surveys.	3
Survey administration	7a	Provide information on modes of questionnaire administration, including the type and number of contacts, the location where the survey was conducted (e.g., outpatient room or by use of online tools, such as SurveyMonkey).	3-4
	7b	Provide information of survey’s time frame, such as periods of recruitment, exposure, and follow-up days.	4
	7c	Provide information on the entry process:<br>–>For non-web-based surveys, provide approaches to minimize human error in data entry.	5

		–>For web-based surveys, provide approaches to prevent “multiple participation” of participants.	
Study preparation	8	Describe any preparation process before conducting the survey (e.g., interviewers’ training process, advertising the survey).	N/A
Ethical considerations	9a	Provide information on ethical approval for the survey if obtained, including informed consent, institutional review board [IRB] approval, Helsinki declaration, and good clinical practice [GCP] declaration (as appropriate).	4
	9b	Provide information about survey anonymity and confidentiality and describe what mechanisms were used to protect unauthorized access.	4
Statistical analysis	10a	Describe statistical methods and analytical approach. Report the statistical software that was used for data analysis.	4
	10b	Report any modification of variables used in the analysis, along with reference (if available).	4
	10c	Report details about how missing data was handled. Include rate of missing items, missing data mechanism (i.e., missing completely at random [MCAR], missing at random [MAR] or missing not at random [MNAR]) and methods used to deal with missing data (e.g., multiple imputation).	5
	10d	State how non-response error was addressed.	N/A
	10e	For longitudinal surveys, state how loss to follow-up was addressed.	N/A
	10f	Indicate whether any methods such as weighting of items or propensity scores have been used to adjust for non-representativeness of the sample.	N/A
	10g	Describe any sensitivity analysis conducted.	N/A
**Results**			
Respondent characteristics	11a	Report numbers of individuals at each stage of the study. Consider using a flow diagram, if possible.	4
	11b	Provide reasons for non-participation at each stage, if possible.	N/A
	11c	Report response rate, present the definition of response rate or the formula used to calculate response rate.	N/A
	11d	Provide information to define how unique visitors are determined. Report number of unique visitors along with relevant proportions (e.g., view proportion, participation proportion, completion proportion).	3
Descriptive results	12	Provide characteristics of study participants, as well as information on potential confounders and assessed outcomes.	3-8
Main findings	13a	Give unadjusted estimates and, if applicable, confounder-adjusted estimates along with 95% confidence intervals and p-values.	4-8
	13b	For multivariable analysis, provide information on the model building process, model fit statistics, and model assumptions (as appropriate).	4
	13c	Provide details about any sensitivity analysis performed. If there are considerable amount of missing data, report sensitivity analyses comparing the results of complete cases with that of the imputed dataset (if possible).	10-11

**Discussion**			
Limitations	14	Discuss the limitations of the study, considering sources of potential biases and imprecisions, such as non-representativeness of sample, study design, important uncontrolled confounders.	10-11
Interpretations	15	Give a cautious overall interpretation of results, based on potential biases and imprecisions and suggest areas for future research.	10-11
Generalizability	16	Discuss the external validity of the results.	10-11
**Other sections**			
Role of funding source	17	State whether any funding organization has had any roles in the survey's design, implementation, and analysis.	10
Conflict of interest	18	Declare any potential conflict of interest.	10
Acknowledgements	19	Provide names of organizations/persons that are acknowledged along with their contribution to the research.	10
